# Comprehensive Flux Modeling of *Chlamydia trachomatis* Proteome and qRT-PCR Data Indicate Biphasic Metabolic Differences Between Elementary Bodies and Reticulate Bodies During Infection

**DOI:** 10.3389/fmicb.2019.02350

**Published:** 2019-10-15

**Authors:** Manli Yang, Karthika Rajeeve, Thomas Rudel, Thomas Dandekar

**Affiliations:** ^1^Department of Bioinformatics, Biocenter, University of Würzburg, Würzburg, Germany; ^2^Department of Microbiology, Biocenter, University of Würzburg, Würzburg, Germany; ^3^Department of Biomedicine, Aarhus University, Aarhus, Denmark; ^4^European Molecular Biology Laboratory, Computational Biology and Structures Program, Heidelberg, Germany

**Keywords:** *Chlamydia trachomatis*, metabolic modeling, metabolic flux, infection biology, elementary body, reticulate body

## Abstract

Metabolic adaptation to the host cell is important for obligate intracellular pathogens such as *Chlamydia trachomatis* (*Ct*). Here we infer the flux differences for *Ct* from proteome and qRT-PCR data by comprehensive pathway modeling. We compare the comparatively inert infectious elementary body (EB) and the active replicative reticulate body (RB) systematically using a genome-scale metabolic model with 321 metabolites and 277 reactions. This did yield 84 extreme pathways based on a published proteomics dataset at three different time points of infection. Validation of predictions was done by quantitative RT-PCR of enzyme mRNA expression at three time points. *Ct*’s major active pathways are glycolysis, gluconeogenesis, glycerol-phospholipid (GPL) biosynthesis (support from host acetyl-CoA) and pentose phosphate pathway (PPP), while its incomplete TCA and fatty acid biosynthesis are less active. The modeled metabolic pathways are much more active in RB than in EB. Our *in silico* model suggests that EB and RB utilize folate to generate NAD(P)H using independent pathways. The only low metabolic flux inferred for EB involves mainly carbohydrate metabolism. RB utilizes energy -rich compounds to generate ATP in nucleic acid metabolism. Validation data for the modeling include proteomics experiments (model basis) as well as qRT-PCR confirmation of selected metabolic enzyme mRNA expression differences. The metabolic modeling is made fully available here. Its detailed insights and models on *Ct* metabolic adaptations during infection are a useful modeling basis for future studies.

## Introduction

*Chlamydia trachomatis* (*Ct*) is one of the medically significant gram-negative species in the genus *Chlamydia*, an obligate intracellular pathogen in humans (Subtil and Dautry-Varsat, 2004). *Ct* particularly infects host epithelial cells and causes human diseases worldwide. It causes over 90 million trachoma cases annually by ocular infection and is responsible for 131 million sexually transmitted infection cases (STI) (Senior, 2012). *Ct* is also reported to contribute to cervical squamous cell carcinoma and co-infection with Neisseria and HIV (Chesson and Pinkerton, 2000; Belland et al., 2003).

*Ct* is characterized by a biphasic developmental cycle: the elementary bodies (EBs), an infectious form which is metabolically inactive, and reticulate bodies (RBs) which is a non-infectious form that is metabolically active. After internalization into the host cell, EBs differentiate into RBs in an intracellular vacuole called inclusion body. In the inclusion, RBs replicate and subsequently reorganize back to EBs (Abdelrahman and Belland, 2005). The mature EBs are released by host cell lysis, they target new host cells to start a new replicative life cycle.

*Ct* is well separated from other eubacteria, and its ∼1 Mb genome presents eloquent homology in both gene structure and order compared to other *Chlamydia* strains that infect human and animal hosts (Stephens et al., 1998; Read et al., 2000; Belland et al., 2003; Subtil and Dautry-Varsat, 2004). The reduced genome of *Ct* is reflected in several incomplete metabolic pathways. The pathogen encodes complete pathways, such as the glycolysis pentose phosphate pathway (PPP), the fatty acid biosynthesis and the tricarboxylic acid (TCA) cycle (Stephens et al., 1998). However, several pathways are fragmented. The Krebs cycle is incomplete and lacks citrate synthase, aconitase and isocitrate dehydrogenase (Stephens et al., 1998). The genus *Chlamydia* is auxotroph for nucleotides, in particular as it is not capable to synthesize phosphoribosyl pyrophosphate (PRPP), the gene encoding ribose-phosphate pyrophosphokinase (EC: 2.7.6.1) (Stephens et al., 1998; Xie et al., 2002; Hove-Jensen et al., 2017). In this context, it is puzzling why the genome encodes many enzymes in the purine and pyrimidine metabolic pathways. Chlamydia is also shown to synthesize peptidoglycans (Liechti et al., 2014, 2016) and to generate high-energy intermediates (e.g., ATP) (Subtil and Dautry-Varsat, 2004). For many of the pathways present in *Ct*, it is unknown whether the enzymes involved are active and the pathways are functional. Moreover, how *Ct* accesses required nutrients in the intracellular vacuolar inclusion with its limited permeability for small molecules is still the focus of studies (Subtil and Dautry-Varsat, 2004). Although the whole genome of many *Ct* strains have been sequenced and annotated (Stephens et al., 1998; Harris et al., 2012), little knowledge is available with regard to which metabolic activities of *Ct* operate in the host cell during infection. Moreover, the lack of effective genetic manipulation tools limits the understanding of chlamydial metabolism (Käding et al., 2014).

Many transcriptomics and proteomics studies have focused on the metabolic properties of *Ct* developmental forms during its biphasic life cycle. Transcriptional profiling of *Ct* was performed (Belland et al., 2003; Albrecht et al., 2010). Later, Omsland et al. (2012) used an axenic medium to detect transcriptional activity in *Ct*, but the transcriptome data of RB are not simple to interpret, as *Ct* is alive in the medium but is not as fully metabolically active as in the host cell. Quantitative proteome profiling was successfully performed (Saka et al., 2011; Skipp et al., 2016) but without time courses. Later Østergaard et al. (2016) reported protein profiling with time courses. A study about the biphasic metabolism of environmental chlamydia on *Protochlamydia amoebophila* was reported by RNA sequencing (Konig et al., 2017). In these studies, EB and RB separation is based on centrifugation. The analysis of essential metabolic pathways has not been achieved and we are still relying on the expression data of *Ct* genes or gene clusters.

To understand the biphasic metabolism of *Ct* during infection, we used genome-scale metabolic modeling with metabolic flux analysis (MFA) to study the metabolic difference between EBs and RBs during different infection stages. Extreme pathway modeling is one approach in MFA, which is based on constraints and tolerance to input data. Extreme pathway modeling is often used in microbial engineering and host-pathogen interaction to study metabolic flux changes under different conditions. In this study, we provide the first *Ct* metabolic model based on detailed reannotation using the protein profiling data with time courses from Østergaard et al. (2016). Using proteomics data as constraints, we furthermore analyzed the implied pathway fluxes according to the reconstructed model. The quantitative genome-scale metabolic modeling and MFA reveal metabolic differences of pathways between EB and RB during infection and provide a first complete overview on the metabolic flux changes of *Ct* during infection.

## Materials and Methods

### Genome-Scale Metabolic Network Model (GEM) Reconstruction

A genome-scale metabolic network reconstruction was based on available genome information of the model strain *Chlamydia trachomatis* D/UW-3/CX (accession: NC_000117) (Stephens et al., 1998), according to the metabolic databases KEGG (Kanehisa and Goto, 2000) and BRENDA (Schomburg et al., 2002), NCBI and all available literature. Additional missing enzyme activities for key pathways in addition to the genome annotation were determined by analysis of these data and experimental data (Østergaard et al., 2016). Gap filling for pathways in our model where an enzyme should be present to allow metabolic conversion was based on biochemical knowledge of complete metabolic pathways as well as data from biochemical databases and literature. If there was no data support for *Ct* of such a metabolic activity, the metabolic gap was not filled. Given the set of involved enzymes, all established and implied metabolites were classified into external metabolites (available metabolic sources and sinks for *Ct* during infection) and internal metabolites processed by the enzyme network. Our model provides both the KEGG reaction number and EC number as available, and these are provided in SBML format.

### Metabolic Flux Modeling and Flux Analysis

Extreme pathways (numbering indicated by a “P” for pathways – they are inferred, no metabolomics or metabolite data) were calculated by YANA square (Pfeiffer et al., 1999; von Kamp and Schuster, 2006; Schwarz et al., 2007) and with the internal convex basis algorithm (Kaleta et al., 2006). The metabolic reactions are prepared in a stoichiometric matrix (**S**) with the size of m * n (**S** = m * n). The rows and columns of the matrix correspond to internal metabolites in the model (m) and involved reactions or enzymes (n), respectively. For each column, the entries describe a stoichiometric correlation with involved metabolites. All possible phenotypes are involved in the non-negative linear combinations of the stoichiometric matrix. The solution of the model was based on the steady state of the network, which is described by the equation: S * v = 0. The solution of this equation describes the complete set of different flux modes satisfying the equation. Each flux mode balances all internal metabolites which are involved in this specific flux mode.

After the steady state was calculated, we estimated enzyme activities from the proteomics data and calculated the optimal pathway fluxes in a dynamic state according to the different metabolic phenotypes at different time points of chlamydial infection. The original data we used were measured in Østergaard et al. (2016). In particular, the Supplementary File S1 file of this publication lists all the chlamydial proteins these authors measured and their identification. Their proteomics raw data from mass spectrometry are available at the ProteomeXchange Consortium^1^, data-set PXD003883.

Flux analysis is a mathematical modeling method to quantitatively simulate the flow of metabolites through the metabolic network. It is widely used in genome-scale metabolic network reconstruction (Orth et al., 2010). Metabolic reconstruction denotes here the process of setting up a metabolic model from scratch by starting from genome annotation and biochemical data to build-up (“reconstruct”) a first complete list of all metabolic enzymes, metabolites, and reactions occurring in the organism.

Based on flux constraints, the stoichiometric approach limits the solution space for the system by enumerating all available limiting pathways (called extreme pathways). Their linear combination predicts all available metabolic network states for a given set of enzymes. With experimental data (Østergaard et al., 2016), an optimized solution can be found by calculating the actual flux strengths of the involved pathways by observing that the error to the estimated enzyme activities is minimized.

These optimal pathway fluxes were calculated by using least square fitting. Our focus was the metabolic difference between EB and RB. Least square fitting by the gradient descent method was generated by YANA square to calculate the best-matched pathway fluxes, including identification of enzymes not presented in proteomic data. Extreme pathways shown are stable, balanced, limiting flux modes of the metabolic system. They represent all adaptation and metabolic pathway capabilities the Chlamydia have, even if the *Ct* textbook pathways are partially incomplete (e.g., TCA). Any metabolic state of the metabolic network in *Ct* is a linear combination of these pure pathways balancing all involved internal metabolites. These pathways are solution space limiting and hence called “extreme” pathways (or P for short). On the other hand, these calculated extreme pathways often cover only part of a textbook pathway, in the results section we refer to this situation as “contributing to a textbook pathway,” when only a subset of all enzymes used in the textbook pathway are involved in this extreme pathway. Moreover, the P numbers given in the following always refer to calculated pathways and not to any directly measured metabolites. There is no direct metabolomics data-set available or given in this study. Instead, using the proteome data as basis and qRT-PCR data for validation, we calculate fluxes according to the extreme pathway system. As these are many flux modes and as these extreme pathways are only contributing to textbook pathways but are not identical to them, it is necessary to number them.

### Cell Culture and *Chlamydia* Infection

Human epithelial cells, HeLa229 (ATCC^®^ CCL2.1^TM^) and human endothelial cells, HUVEC cells (ATCC^®^ CRL-1730^TM^) were used for propagating bacteria and for basic experiments. HeLa229 cells were grown in RPMI1640 + GlutaMAX^TM^ (Gibco^TM^ 72400-054) with 10% heat inactivated FCS (Sigma-Aldrich F7524). HUVECs were grown in M200 media with growth supplements LSGS (Invitrogen). *Chlamydia trachomatis* (serovar L_2_/434/Bu) was used in the study. *Chlamydia* was prepared as previously published (Karunakaran et al., 2015). Briefly, *Chlamydia* were grown in HeLa229 cells at a MOI (multiplicity of infection) of 1 for 48 h. The cells were lysed using glass beads (15 mm) for 3 min and centrifuged at 2000 *g* for 10 min to remove the cell debris. The supernatant -containing bacteria was collected and centrifuged at 24,000 *g* for 30 min at 4°C. The pellet was washed and resuspended in SPG buffer [10 mM sodium phosphate (8 mM Na_2_HPO_4_-2 mM NaH_2_PO_4_), 220 mM sucrose, 0.50 mM L-glutamic acid], aliquoted and stored in −80°C. *Chlamydia* EBs and cell lines used in the study were verified to be free of *Mycoplasma* contamination via PCR. The bacteria were titrated to have a MOI of 1 and were used further in the experiments.

### Quantitative Real-Time PCR

RNA was isolated from uninfected and *Chlamydia* infected HeLa229 cells and HUVEC cells using a RNA easy kit (Qiagen, Germany). RNA was reverse transcribed using a Revert Aid First Strand synthesis Kit (Fermentas) according to the manufacturer’s instructions and diluted 1:10 with RNase free water. qRT-PCR was performed as previously described (Karunakaran et al., 2015). Briefly, qRT-PCR reactions were prepared with Quanta SYBR (Quanta Bio) and PCR was performed on a Step One Plus device (Applied Biosystems). Data were analyzed using ΔCt method, Step One Plus software package (Applied Biosystems) and Excel (Microsoft). The endogenous control was Ct16SrRNA. Primers were designed by qPrimer Depot. The primers are listed in Table 1.

**TABLE 1:** Primers used for the PCR analysis.

**Oligo**	**Gene**	**Order**	**Sequence 5′–3′**
CT121	araD	fwd	GTCCGGAGCAGATCTTATTCAT
		rev	TCAAAATGCACAATGATACGATCC
CT750	tktB	fwd	CTAAAGATTCTCGATGGGTGAATA
		rev	TGTAGTGGCTTCTACACCATCA
CT313	tal	fwd	CTCTGACTTTTGTTTTAGATAAGATC
		rev	TTTTAACTAATAAACGTTTCTTATCTCCT
CT505	gapA	fwd	GGAGAAAGAAAGATCCGTTTCTTA
		rev	TCCCATAACAAACGTAGGGACA
CT714	gpdA	fwd	AGAATCTTTCTTTCACATACCACAT
		rev	CGATTTCGCTTAGGAGTAAGC
CT807	plsB	fwd	GATGACCTTAAGAATCCTATTATTTTC
		rev	TACATGAGCTGGGGGTCTGA
CT811	plsX	fwd	CCTCGATGGCTCTAGGATTG
		rev	TCTCGTCAGGATTCACGGAC
CT453	plsC	fwd	GCTAAGTCCGGACTGTTTTCTA
		rev	AATAACAGGGACATTGCCTTTGATA
CT775	tktB	fwd	GGTTAAAGTTTCACGTGCGAC
		rev	AACGATCTCTTCTTTCCCATTTCT
CT821	sucC	fwd	GAGTATTATCTTGCGATCGTCAT
		rev	ATCGGCAATAGGTTGATCCCA
CT822	sucD	fwd	CCGTTTGCTGCAGAAGCCAT
		rev	ATATACCCAGGCATGATGCCA
CT054	sucA	fwd	TGGAGTTTGAAGACGCTCCC
		rev	TGACCCGCCAAGGCTGC
CT055	sucB	fwd	GCGATGCGCAGATTATTGCC
		rev	TTCATTAAATGTCGTTAACATTGCTG
CT376	mdhC	fwd	GCCTATAGCTTTCTATTTTCTCTG
		rev	CATCAAAAGCATCATGCAATGATG
CT855	fumC	fwd	ATGCGGCAAGAGAATGATAGC
		rev	AGCAACAATCATATCCCTGCG
CT238	fabD	fwd	CTGGGATTAGAAATAAGGTGGAA
		rev	ATACACTAACTCCCCTATTACATTA
CT816	glmS	fwd	GGCGTAGTATTTTCATCAGATAC
		rev	ACCTCGTCTTCTCCTAATCCTA
CT183	pyrG	fwd	AAGTATACGTGACCGACGATG
		rev	CTGCGCACGATTGAATGACAT
CT614	folX	fwd	GTCTCAGAACAAGAACGGCATT
		rev	GCCAAAGCCTTTTCTATTTTTTCC
CT613	folP	fwd	GGGAAACGCAGCTATCTCCA
		rev	ACGATACGGACATAAAGAAGCC
CT612	folA	fwd	CATTCGAAATCATCCCATCATTATG
		rev	TGTGACAAAACAAGCTTTCAGAAG
CT378	pgi	fwd	AGGATGCTTATCTGAAGAGCGT
		rev	CTTCTATACGATTCATGATGGCG
CT205	pfkA1	fwd	CTCTGTTGCAGAACTATTTCCATG
		rev	CTGTGTAGCAATCCTAAGATGAC
CT207	pfkA2	fwd	CCCAGACCTCACTCTTTCAAAAA
		rev	CTCGAATAAGTCCAAGAGGGC
CT332	pykF	fwd	GGCCGGACCATCGCTATTC
		rev	GAGAGTAAGACGATCCCCAG

## Results

### Reconstruction of the Genome-Scale Metabolic Model of *Chlamydia trachomatis* D/UW-3/CX

**FIGURE 1:**
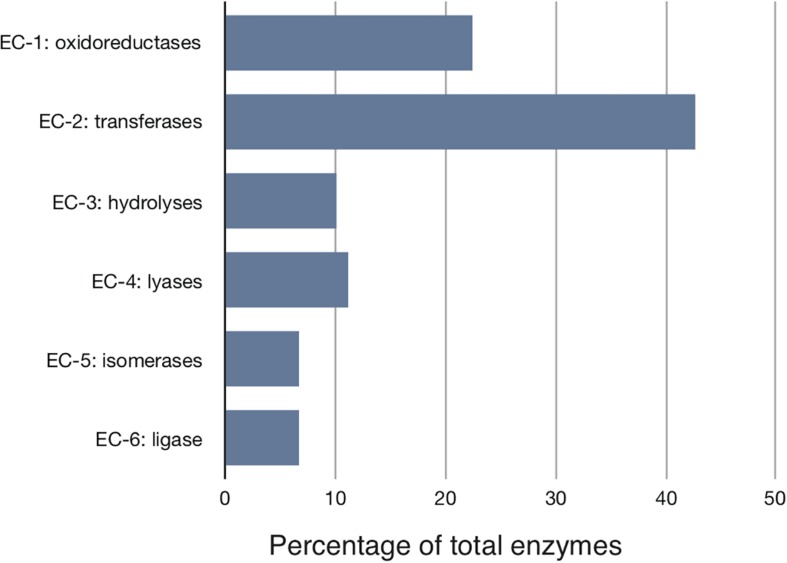
Distribution of the major enzyme classes in Chlamydial metabolism. Enzymes involved in the reconstructed model are classified according to the Enzyme Commission (EC) classification. Shown is the percentage of enzymes in each EC class. Transferases represent over 40% of all activities. *Ct* modifies host building blocks for incorporation into its own metabolism.

**FIGURE 2:**
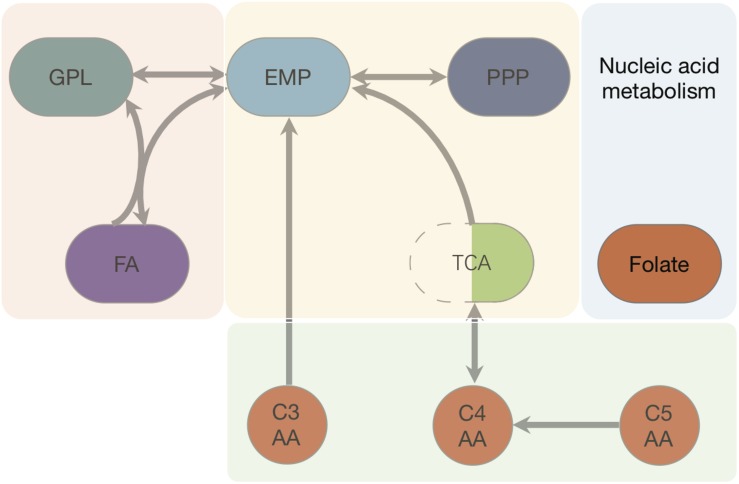
Pathways and fluxes in *Ct*. Sink and source metabolites are shown, the different pathways present in *Ct* are given as complete and incomplete lines. Approximate flux strength are indicated. The pathway with the strongest flux is shown as blue thick line (path from pyruvate → acetyl–CoA → malonyl–CoA = malonyl–ACP). A detailed vector graphic is given in supplement, showing all internal and external metabolites and flux strengths quantitatively.

We initially established a genome-scale model of *C. trachomatis* based on genome annotation, biochemical databases (KEGG), and manual curation. It contains 321 unique metabolites and 277 chemical reactions. Theoretically, there could be more metabolites and more reactions: This depends on the number of enzymes identified from all available sources for this organism (here: *Ct*) and the known compounds these enzymes involve. However, we focus here on the primary metabolism and consider only those reactions relevant to the primary metabolism. Nevertheless, an extensive check of all available data is necessary for an accurate estimate (this process is called “metabolic reconstruction”):

We started with the well-annotated genome sequence of *Chlamydia trachomatis* D/UW-3/CX (accession: NC_000117) and compared the model to the available proteomics dataset for the curation of the annotation. The curation involved monitoring of any obvious pathway gaps and re-examining the function of the involved enzymes (full annotation given in Supplementary File S1). Transferases and oxidoreductases together constitute 64% in the metabolic network, with 72 and 37 enzymes respectively. Isomerases and ligases are less present (12 for each of them; Figure 1). Central subsystems in the primary metabolism of *Ct* include the metabolism of carbohydrates, energy, nucleotides, amino acids, lipids, glycans, cofactors, and vitamins. In order to maintain the accuracy of the model, some gaps were filled manually and poorly validated reactions (according to all available data; see Materials and Methods) were removed. The result is a first genome-scale metabolic model of the central and primary metabolism of *Ct.* The model is described in SBML (Level 2) format (Supplementary File S2), a simplified overview is visualized in Figure 2 and summary features are provided in Table 2.

**TABLE 2:** Features of *Ct* genome and metabolic network model.

**Genome characteristics**	
Genome size	1.04 Mb
Total ORFs	935
Proteins	887
**Metabolic model characteristics**	
Metabolic enzymes	171
Metabolic reactions^a^	277
Metabolites	321
Extreme pathways	84

*^*a*^As some enzymes are involved in more than one reaction, this number is higher than the number of metabolic enzymes.*

### Metabolic Fluxes in Chlamydia Based on Extreme Pathways

The model with full annotation was next transformed into a stoichiometric matrix considering all enzymes and their reversibility while differentiating between internal metabolites and metabolic sources and drains (external metabolites). This matrix (Supplementary File S3) was next used to calculate extreme pathways using flux balance analysis using a proteome dataset as basis. 84 extreme pathways (Supplementary File S4) were calculated based on this stochiometric matrix input. From the dataset relative flux strengths for each of these pathways were calculated (Supplementary File S5) according to Kaleta et al. (2006), using the proteomics data by Østergaard et al. (2016) as an estimate for each enzyme activity (see section Materials and Methods). Supplementary File S6 visualizes the resulting quantitative fluxes as calculated (Supplementary File S5). The pathway with the strongest flux strength in the whole system starts from pyruvate (the blue line visualizes this strongest flux in Supplementary File S6). This is turned into acetyl-CoA, then into malonyl-CoA, and ends with malonyl-ACP. Three points of time were then chosen during the infection, which were 20 h post infection (hpi), 40 hpi and the lysis phase, respectively. The experimental dataset input for flux analysis was from the reported proteomics dataset (Østergaard et al., 2016). For each data group, there were given data of normalized iBAQ values on the protein abundance of EB and RB after their separation by centrifugation. As the ratio between EB and RB in the original experiment was not provided as data, we combined EB and RB’s protein normalized iBAQ data, as these were quantitative measurements (Østergaard et al., 2016).

Considering the EB + RB data together at each time point allows us to describe the general features of the *Ct* at that time. Roughly at 8 hpi, the EBs start to transform to RB. At 20 hpi, RBs dominate compared to EBs. During the time from 36 hpi to 40 hpi, the RB form of *Ct* converts back to EB. Hence, at this stage, the number of RB is supposed to decrease and the number of EB’s to increase. The comparison of the quantitative fluxes of EB, RB, and EB + RB at three different time points is a very dense, data-rich figure.

It is hence shown in detail only as Supplementary File S7 (y-axis: results of the nine different flux calculations as a heat map indicating flux strength (vertical bar on top shows intensities; x-axis gives all 84 extreme pathways calculated with their P numbers; Supplementary File S5 lists all the enzyme reactions involved in each extreme pathway according to the same numbering; fluxes are visualized in Supplementary File S6). In the following we summarize the main results of these calculations:

The extreme pathways calculated are all stable, balanced limiting flux modes of the metabolic system (see section Materials and Methods). They reveal which adaptation and metabolic pathway capabilities Chlamydia have, even if textbook pathways are partly incomplete (e.g., TCA). Any metabolic state of this network is a linear combination of these pure (solution space limiting and hence called “extreme”) pathways (see P numbers). On the other hand, these calculated pathways frequently cover only part of a textbook pathway, they only contribute to it (see above). From the modeling, we can infer that there are a few central pathways which carry most of the metabolic flux during infection, such as the strongest flux path from pyruvate to malonyl-ACP (pathway number P65), followed by the downstream part of glycolysis (pathways number P5, P34, P83), upstream part of fatty acid biosynthesis (extreme pathways number P66 and P67), and a calculated flux pathway involved in biotin metabolism (P65). Cysteine biosynthesis also presents a strong flux as calculated from the proteome data (P16). Many reactions in nucleotide metabolism present strong intensities (extreme pathways P8-P15, P31, P32, P40-45). Only medium activity was inferred from the proteome data for glycolysis and glycerophospholipid pathway (P34, P83), PPP (P84), and folate biosynthesis or conversion (P33, P51, P55). Always only very low activity was inferred for fatty acid biosynthesis (P66, P67), phenylalanine and tyrosine biosynthesis (P53, P54), and porphyrin and chlorophyll metabolism (P29).

So, based on the proteome data by Østergaard et al. (2016), the relative flux strengths were calculated. The results apply to the relative pathways within one cell, be it EB or RB. If we instead want to compare EB versus RB, we need experimental approaches, such as strong separation between EB and RB (see section Discussion). The proteome data by Østergaard et al. (2016) give a differential picture on the involved enzymes and their activity. The authors found activity of RB > EB for 90 proteins and RB < EB for 51 proteins. However, protein abundance is only an indirect measurement of enzyme activity. As there also may be technical separation problems, we considered and always give in addition the total proteome data available (EB + RB).

#### Central Metabolic Pathways

The central metabolic pathways present in *Ct* are summarized in Table 3. They are all present in *Ct* and represented in the metabolic model with their respective fluxes. Pathways involve the PPP, the glycolysis Emden-Meyerhof-Parnass pathway; EMP) and glycerol-phospholipid (GPL) pathway as well as fatty acid (FA) biosynthesis. Moreover, there is the citric acid or tricarboxylic acid (TCA) cycle and a cycle formed by two phosphofructokinases (pfkA_1 and pfkA_2).

**TABLE 3:** Extreme pathways in central metabolism^∗^.

**Pathway**	**Reaction**
PPP (P84)	6PGn + 2G6P + 2GDP + 2H2O + 5NAD + + 4NADP + + Orthophosphate → 6PGL + 4CO2 + 2GTP + 9H + + 5NADH + 4NADPH + Pyr + Ru5P
EMP/GPL (P83)	2P + 2Acyl–CoA + CTP + 2G6P + 4GDP + NAD + + NADPH → 2CoA + Cytidine + 4GTP + H2O + NADH + NADP + + Orthophosphate + PGP + 2Pyr
pfkA (P34)	ATP + Orthophosphate → 2P + ADP
TCA (P46)	2ADP + FAD + 2Glu + 2H2O + 4NAD + + 2Orthophosphate + Quinone → 2ATP + 2Asp + FADH2 + 4H + + Hydroquinone + 4NADH
FA1 (P66)^∗∗^	8ATP + 2Acetyl–ACP + 18H + + 18HCO3– + 6NAD + + 26NADPH + 18Pyr → 18ADP + 36CO2 + 4FA_ex + 18H2O + 6NADH + 26NADP + + 18Orthophosphate
FA2 (P67)^∗∗^ EMP/GNG (P5)	2ACP + 18ATP + 16H + + 18HCO3– + 8NAD + + 26NADPH + 20Pyr → 18ADP + 38CO2 + 4FA_ex + 18H2O + 8NADH + 26NADP + + 18Orthophosphate 2P + G6P + 4GDP + 2NAD + + 2P + G6P + 2NAD + + Orthophosphate = 4GTP + 2H + 2H2O + 2NADH + 2Pyr

*^∗^PPP, pentose phosphate pathway; EMP/GPL, glycolysis and glycerophospholipid biosynthesis; pfkA, 6-phosphofructokinase 1 (pfkA_1, EC 2.7.1.11) and diphosphate-fructose-6-phosphate 1-phosphotransferase (pfkA_2, EC 2.7.1.90) formed cyclic pathway; TCA, tricarboxylic acid cycle; FA1 and FA2, both are fatty acid biosynthesis. EMP/GNG, glycolysis and gluconeogenesis. ^∗∗^In fatty acid biosynthesis, FA1 utilizes 3-oxoacyl-ACP synthase II (FabF, CT_770, EC 2.3.1.179) on the step from malonyl-ACP to acetoacetyl-ACP, while FA2 use both FabF and 3-oxoacyl-ACP synthase III (FabH, CT_239, EC 2.3.1.180) for the same step. ACP, acyl carrier protein.*

**FIGURE 3:**
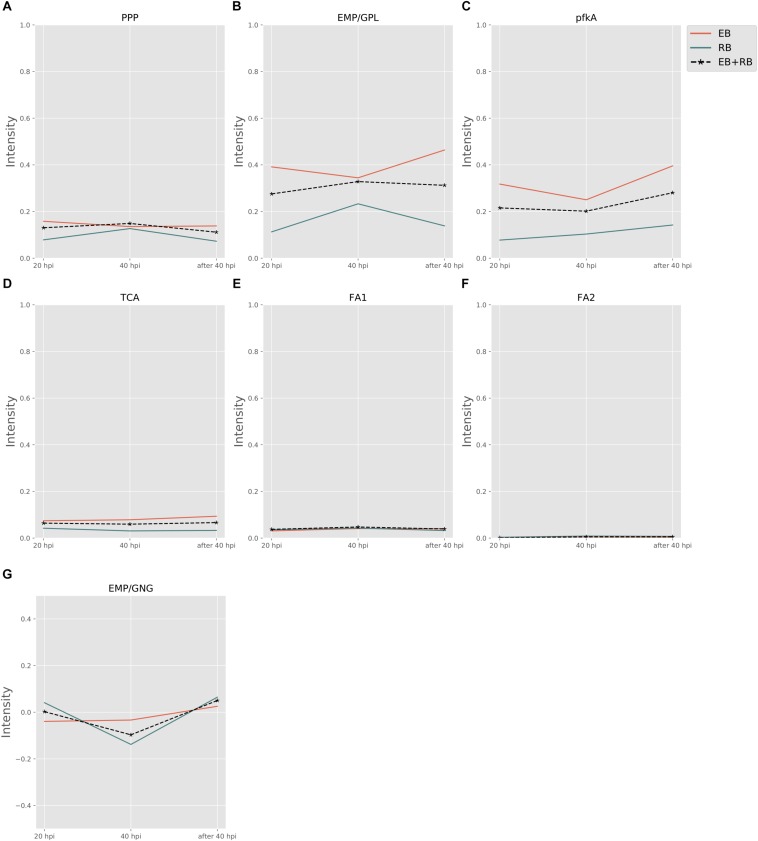
Pathway activity changes of central metabolism in *Ct* during infection. EB flux changes are given by a red solid line and RB flux changes are shown as green solid line; the average flux change during infection (combining EB and RB as present during infection in the host cells) is given as black dashed line. The time course indicates the variation over infection from 20 hpi, 40 hpi and several hours (about 8 h) later after 40 hpi respectively. Flux intensities are normalized between zero and one (fully active). **(A)** pentose phosphate pathway (PPP); **(B)** glycolysis and glycerophospholipid metabolism (EMP/GPL); **(C)** a cyclic pathway formed by 6-phosphofructokinase 1 and diphosphate-fructose-6-phosphate 1-phosphotransferase (pfkA); **(D)** tricarboxylic acid cycle (TCA); **(E,F)** fatty acid biosynthesis (FA1) and (FA2); and **(G)** gluconeogenesis (GNG).

Figure 3 shows the pathway strength of the central metabolism of *Ct* during infection. Central carbohydrate pathways, such as PPP, EMP/GPL, and pfkA presented higher intensities in both EB and RB when compared to the flux carried by TCA and FA. EB shows carbohydrate pathway activity, in particular regarding PPP, EMP/GPL, and to a lesser extent even TCA. As far as can be inferred from the data provided by Østergaard et al. (2016), in RB the pathway activity distribution shifts toward EMP/GPL. The EMP/GPL pathway was much more active than the average central metabolism with an average intensity of 0.40 in EB and 0.16 in RB (Supplementary File S5). This means, in RB the flux of glycolysis is on and flows also into the GPL synthesis, which is helpful for replication. In contrast, in EB we have low metabolic activity but still some active metabolism (Grieshaber et al., 2018). Bearing this in mind, in this residual metabolism there is a sizeable fraction used for glycolysis and then channeled into GPL synthesis according to our data. This suggestion from the metabolic modeling was subsequently supported by individual measurements of the enzyme transcripts (see RT-PCR data below).

Phosphofructokinase, as the pacemaker of glycolysis, presented an average intensity of 0.32 in EB and 0.11 in RB (Supplementary File S5). This suggests glycolysis and GPL biosynthesis, which are more active in EB than in RB, are of vital importance in *Ct* metabolism.

Besides the dominant glycolysis, *Ct* has the capability of synthesizing its own phospholipids and fatty acids (though in low intensity) in both EB and RB groups (Figure 3). *Ct* was reported to utilize host fatty acid for phospholipid synthesis (Yao et al., 2015), and to accumulate lipid droplets (LDs) (Kumar et al., 2006; Cocchiaro et al., 2008). Also, the pathway exhibiting the strongest strength contains the downstream of glycolysis and the upstream of fatty acid synthesis. The whole pathway of biosynthesis of fatty acid is inferred to be almost inactive, with average flux intensities below 0.04 and 0.01 in FA1 and FA2 respectively in both EB and RB groups. This suggests that *Ct* may acquire acyl-CoA from host LD to synthesize its own phospholipids and utilize host fatty acid directly when forming its outer membrane.

The intensity of TCA was below the total average for both EB and RB, with a value of 0.08 and 0.04 (Supplementary File S5), and presented a slight reduction in RB. This may be because *Ct* could take the energy source (e.g., ATP) directly from the host cell rather than rely on producing the energy independently. TCA is not much active in EB when compared to the other pathways and even less active in RB. This may also suggest that RB may take more energy from the host. Fatty acid biosynthesis has two pathways, FA1 (using 3-oxoacyl-ACP synthase II) and FA2 (using 3-oxoacyl-ACP synthase II and III). FA1 was observed to have an intensity of less than 0.04 (Supplementary File S5) on average (roughly only 10% of the flux of glycolysis in EB and 20% in RB). FA2 was almost not activated at all, though with values below 0.01 (Supplementary File S5) in both EB and RB. Nevertheless, the FA biosynthesis still shows metabolic activities during development for both EB and RB, though it is hard to tell the difference in the metabolic level between EB and RB during the time elapsing.

Not only glycolysis, but also gluconeogenesis was observed in our analysis. In Figure 3G, during the infection from 20 to 40 hpi and the lysis phase, EB steadily went from gluconeogenesis to glycolysis. RB, whose variation trend matches EB + RB’s general trend, converted from glycolysis to gluconeogenesis, and finally transformed again to glycolysis. One possibility suggested from our study is that RB could take up more carbon sources (e.g., G6P) from the host cell at 20 hpi. However, then it will probably end up in cell wall synthesis, as data by Mehlitz et al. (2017) show, where RB uses glucose-6-phosphate for cell wall biosynthesis and not for energy production. Once the transformation from RB to EB begins, carbon sources are stored for further usage. Though the values for RB (−0.14) were over three times higher than EB (−0.03) at 40 hpi (Supplementary File S5), it is still hard to conclude which form prefers gluconeogenesis because of the unknown quantitative ratio between EB and RB. Nevertheless, there is preference of glycolysis both in EB and RB at 40 hpi post.

#### Folate Biosynthesis Pathways

**FIGURE 4:**
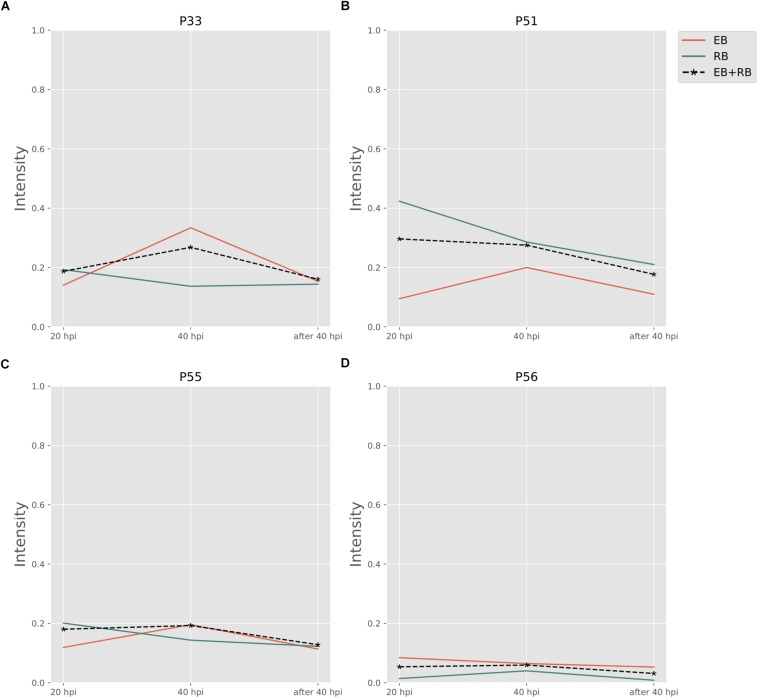
Pathway activity changes of Folate biosynthesis in *Ct* during infection. **(A)** P33; **(B)** P51; **(C)** P55; and **(D)** P56 are all extreme pathways involved in synthesizing folate. EB flux changes are given by a red solid line and RB flux changes are shown as green solid line; the average flux change during infection (combining EB and RB as present during infection in the host cells) is given as black dashed line. The time course indicates the variation over infection from 20 hpi, 40 hpi and several hours (about 8 h) later after 40 hpi respectively. Flux intensities are normalized between zero and one (fully active).

Folate pathways are also central pathways for *Ct*. Contributions to folate biosynthesis come from four extreme pathways (Figure 4; extreme pathways P33, P51, P55, and P56). P33 and P51 pathways convert glutamate and 4-aminobenzoic acid (PABA) to glycolaldehyde and folate (see Table 4). The flux strengths of both pathways were enriched above average for all pathways, which indicates the significance of folate biosynthesis in *Ct*. The difference between P33 and P51 extreme pathways was the amount of ATP and water consumed. Less ATP was required in extreme pathway P33, which is more activated in EB than in RB. In contrast, P51 displayed higher activation in RB than EB when more ATP is available. The variable trend of EB + RB presented a similar result, which increased first and then decreased like EB in P33, and steadily went down during the infection which fits for RB in P51. The ATP generated in P51 but not in P33 relied on the ATP:AMP phosphotransferase (reaction: ATP + AMP = 2ADP). This suggests RB relies more on ATP consumption when it is easier to utilize from both the host and the self-production by activated nucleotides metabolism. There are other folate-related pathways with less ATP consumption in P55 and P56 albeit with lower intensity. P55 was the transformation among folate, tetrahydrofolate, and di-hydro-folate. And P56, although not as active as P33 and P51, could be an alternative pathway for folate biosynthesis, especially for EB.

**TABLE 4:** Extreme pathways in folate biosynthesis.

**Pathway**	**Reaction**
P33^a^	2ATP + 2GTP + 2Glu + NAD + + NADP + + 2Orthophosphate + 2PABA → 4(2P) + 2ADP + 2Folate + 2Formate + 2Glycolaldehyde + 2H^+^ + NADH + NADPH
P51^b^	6ATP + 2GTP + 2Glu + 2H2O + NAD + + NADP + + 2Orthophosphate + 2PABA → 2P + 6ADP + 2Folate + 2Formate + 2Glycolaldehyde + 2H^+^ + NADH + NADPH
P55^c^	Folate → THF = DHF = Folate
P56^d^	2(5–Formyl–THF) + 2ATP + 2Gly + 2H2O + NAD + + NADP + → 2ADP + 2Folate + 2H^+^ + NADH + NADPH + 2Orthophosphate + 2Ser

**Reaction involved based on KEGG reaction number: ^*a*^R03504, R03066, R02235, R00425, R02237, R04621, R11072; ^*b*^R00127, R03504, R03503, R03067, R02235, R00425, R02237, R04621, R011071; ^*c*^R02235, R00937, R00936; ^*d*^R02235, R00936, R00945, R01200, R02301.**

Finally, the P33 and P51 pathways suggest glutamate is in great demand for *Ct* or at least in the folate pathways. Also, it is likely that folate metabolism is tightly correlated with the availability of carbon/nitrogen sources and energy production in *Ct* (Fan et al., 1992; Adams et al., 2014).

### Amino Acid Metabolism

#### Cysteine Metabolism

**FIGURE 5:**
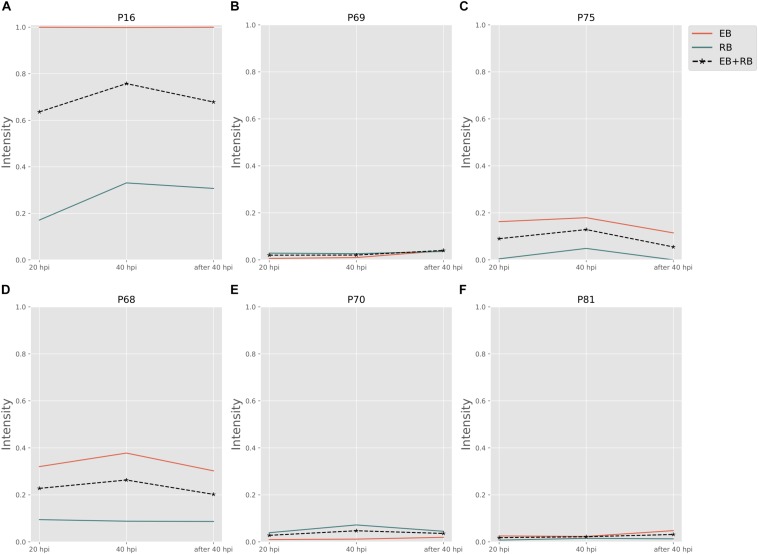
Cysteine and other amino acid associated activity changes in *Ct* during infection. **(A–C)** Are cysteine biosynthesis pathways; **(D–F)** are cysteine consumptions. EB flux changes are given by a red solid line and RB flux changes are shown as green solid line; the average flux change during infection (combining EB and RB as present during infection in the host cells) is given as black dashed line. The time course indicates the variation over infection from 20 hpi, 40 hpi and several hours (about 8 h) later after 40 hpi respectively. Flux intensities are normalized between zero and one (fully active).

Biosynthesis of cysteine is very important for forming the outer membrane of EB, because *Ct* contains the type three secretion system (T3SS) in the outer membrane. In order to compose T3SS, cysteine is assumed to accumulate more in the infectious form (Betts-Hampikian and Fields, 2011). In our results, intensities of cysteine synthesis pathways (Figures 5A–C) on average are higher than those of cysteine consuming pathways (Figures 5D–F). With a clear difference in fluxes, it is obvious that the formation of cysteine is much more intense in EB rather than in RB. The intensities are two to five times higher in EB when compared to RB. Our data support the hypothesis that the cysteine-rich outer membrane is constructed in EB.

#### Peptidoglycan Biosynthesis

The P70 pathway (Table 5) is involved in the biosynthesis of peptidoglycan (PG). The *Mur* genes for PG synthesis are sensitive to penicillin. However, the absence of PG in *Ct* was quite unusual. New cell wall labeling methods helped reveal PG for the first time (Pilhofer et al., 2013; Liechti et al., 2014). P70 flux pathway, though with weak intensity, was inferred from the calculation to be active only in RB (Figure 5E). Peptidoglycan synthesis is also known to occur only in RB:

**TABLE 5:** Cysteine and other amino acid associated extreme pathways.

**Pathway**	**Reaction**
P16^a^	Cysteinyl–glycine + H2O → Cys + Gly
P68^b^	4-Methylthio–2-oxobutanoate + Cys → Mercaptopyruvate + L-Methionine
P69^c^	Cysteate + 3H + + Mercaptopyruvate + 3NADPH → Cys + 2H2O + 3NADP + + Pyr + Sulfide
P70^d^	7ATP + 2Ala + Asp + Cys + GlcN6P + Glu + H + + NAD + + 2NADPH + 2Pyr + UDP–MurNAc + Undecaprenol → 2P + 7ADP + CO2 + H2O + Mercaptopyruvate + NADH + 2NADP + + 6Orthophosphate + Peptidoglycan
P75^e^	2ADP + 2Asp + GTP + ITP + 2Mercaptopyruvate → 2ATP + 2CO2 + 2Cys + GDP + IDP + 2Pyr
P81^f^	2P + 3ADP + 3Cys + 2G6P + 12GTP + 3H + + 3NADPH + 6Pyr → 3ATP + 3CO2 + 12GDP + 3H2O + 3Mercaptopyruvate + 3NADP + + 13Orthophosphate + 3Phe

**Reactions involved based on KEGG reaction number: ^*a*^R00899; ^*b*^R07396, R00895; ^*c*^R00858, R02433, R00895, R04861; ^*d*^R00014, R03270, R02569, R07618, R00416, R05332, R02060, R03193, R02783, R02788, R01150, R04617, R05630, R05032, R00480, R02291, R10147, R04199, R07613, R02735, R00895, R05626; ^*e*^R00431, R00200, R00895, R00355; ^*f*^R01056, R01529, R02740, R01070, R01830, R01827, R01641, R01015, R02073, R00330, R01826, R03083, R03084, R02413, R02412, R03460, R01714, R00694, R00895, R01715, R01373.**

#### Uptake of Glutamate and Glutamine

The TCA cycle in *Ct* is incomplete and presents a modified horse shoe form providing substrate for amino acid synthesis but with a low energy yield. The reason for this is the lack of genes encoding citrate synthase, aconitate hydratase and isocitrate dehydrogenase. Substrate enters into this incomplete TCA cycle by 2-oxoglutarate and turns out to oxaloacetate. With the appearance of aspartate transaminase (EC 2.6.1.1), the oxaloacetate could exchange with aspartate accompanied by barter between glutamate and 2-oxoglutarate. However, the flux prefers to drive from glutamate to aspartate [Figure 3D and Table 3, TCA (P46)]. It is possible that the anaplerosis shown in Figure 3D is commonly completed in almost all aerobic organisms. Nevertheless, because of the low flux strengths, this unfeasible cycle is not significantly responsible for gaining chemical energy in the form of ATP.

In most microorganisms, glutamine synthetase (EC 6.3.1.2), glutaminase (EC 3.5.2.1), and glutamate synthase (EC 1.4.1.13/1.4.1.14) are important for switching between glutamate and glutamine. Normally, glutamine could be converted to glutamate after taken from the environment or host cell. Without encoding genes for these enzymes, *Ct* presents glutamine-fructose-6-phosphate transaminase (glmS, EC 2.6.1.16), whose protein product converts glutamine and fructose 6-phosphate to glutamate and glucosamine 6-phosphate. The existence of CTP synthase (pyrG, EC 6.3.4.2), should allow to transform glutamate into glutamine. PyrG seems not to be involved in long essential pathways such as glmS, as no full pyrimidine metabolism is formed in *Ct*. Both glmS and pyrG were detected with expressions in the proteomics data used (Østergaard et al., 2016).

Although glutamate and glutamine are both necessary in the metabolism of *Ct*, it is still unknown whether *Ct* imports only glutamine or both glutamine and glutamate from the host cell. Interestingly, three glutamate-related transporters, CT_216, CT_230 and CT_401 are encoded in the genome, and both CT_216 and CT_401 are expressed in the protein level (Østergaard et al., 2016). However, glutamine transporters are not confirmed yet.

#### Fragmented Pathways for Purine and Pyrimidine Synthesis

**FIGURE 6:**
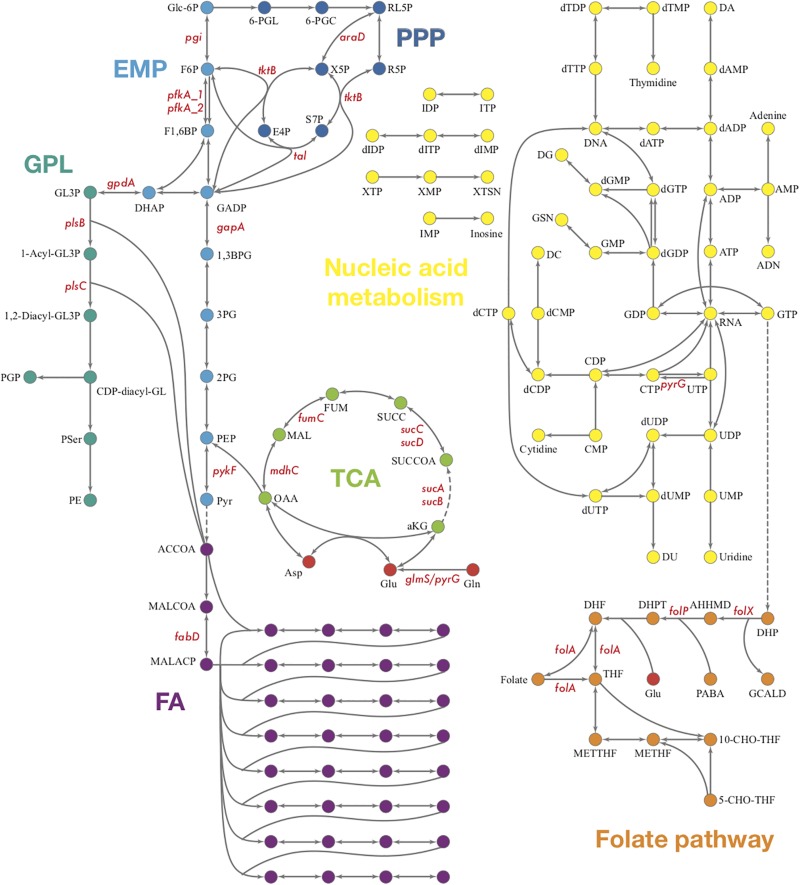
Overview of *Ct* central metabolism. The enzymes labeled in red were validated in their activity of expression by RT-qPCR. EMP, glycolysis; PPP, pentose phosphate pathway; GPL, glycerophospholipid metabolism; GNG, gluconeogenesis; TCA, tricarboxylic acid cycle; FA, fatty acid biosynthesis. *araD*, ribulose-phosphate 3-epimerase; *tktB*, transketolase; *tkl*, transketolase; *pgi*, glucose-6-phosphate isomerase; *pfkA_1*, 6-phosphofructokinase; *gapA*, glyceraldehyde 3-phosphate dehydrogenase; *pykF*, pyruvate kinase; *pfkA_2*, pyrophosphate–fructose-6-phosphate 1-phosphotransferase; *gpdA*, glycerol-3-phosphate dehydrogenase (NAD(P) +); *plsB*, glycerol-3-phosphate O-acyltransferase; *plsX*, glycerol-3-phosphate O-acyltransferase; *plsC*, 1-acyl-sn-glycerol-3-phosphate acyltransferase; *mdhC*, malate dehydrogenase; *fumC*, fumarate hydratase class II; *sucC*, succinyl-CoA synthetase beta subunit; *sucD*, succinyl-CoA synthetase alpha subunit; *sucA*, 2-oxoglutarate dehydrogenase; *sucB*, 2-oxoglutarate dehydrogenase; *fabD*, Malonyl CoA-acyl carrier protein transacylase; *glmS*, glutamine-fructose-6-phosphate aminotransferase; *pyrG*, CTP synthase; *folX*, dihydroneopterin aldolase; *folP*, dihydropteroate synthase; *folA*, dihydrofolate reductase.

The biosynthesis of phosphoribosyl pyrophosphate (PRPP), an important precursor in nucleotide biosynthesis, is not available in *Ct*, due to the lack of PRPP synthase based on the genome (Stephens et al., 1998). Whether PRPP could be acquired through other methods of synthesis or from the host cell through a membrane transporter is still unknown. Theoretically, the central purine and pyrimidine metabolism would not be present in *Ct* for the lack of PRPP. However, many genes encoding enzymes involved in nucleotide acid metabolism are present in *Ct*’s genome which forms theoretical pathways (Figure 6). Also, some of these enzymes are detected in the proteomics and shown to be strongly up-regulated when two or three reactions form small fragments of pathways in our results (Supplementary File S5).

#### Biological Validation of *in silico* Results in Cell Lines

**FIGURE 7:**
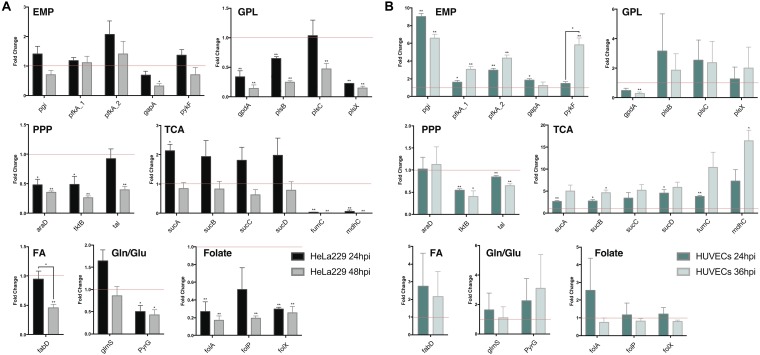
Quantitative RT-PCR analysis of gene expression in major metabolic pathways of *Ct* during infection in HeLa229 cell lines and HUVECs cell lines. The gene expression of key metabolic enzymes in *Ct* (shown in Figure 6) was measured at 24 hpi and 48 hpi for **(A)** (HeLa229), and 24 hpi and 36 hpi for **(B)** (HUVECs). Each gene was quantified by three independent experiments with the mean values (± SEM) compared to the expression at 12 hpi shown as a control (red line). Statistical analysis was performed by SEM and Student *t*-test (^∗^*p* ≤ 0.05; ^∗∗^*p* ≤ 0.01). PPP, pentose phosphate pathway; EMP, glycolysis; GPL, glycerophospholipid biosynthesis; TCA, tricarboxylic acid cycle; FA, fatty acid biosynthesis; Gln/Glu, converting glutamine to glutamate; Folate, Folate biosynthesis.

To validate the results of the metabolic model, gene expression of candidate enzymes in *Ct* were selected and examined by RT-qPCR (Table 6). These enzymes were chosen as they cover our metabolic model well and the key changes according to our pathway model are shown in Figure 6 (enzymes shown by red lettering). Quantitative RT-PCR provided the method of choice for us as it is sensitive and fast. The measurements in triplicate are quantitatively accurate, however, as they measure only a marker of enzyme activity, i.e., the amount of mRNA present to synthesize the respective enzyme, they allow us only to validate our flux predictions by corresponding enzyme mRNA expression levels. Nevertheless, regarding bacterial metabolism, and considering the rapid turnover of bacterial enzymes, they are informative to validate our metabolic model in this sense. To achieve even more robustness in our measurements, we compare here measurements in two cellular models, HeLa229 cells and HUVECs cells (Figure 7). Keep also in mind that the flux predictions (all flux data are given in Supplementary File S5) were selected by optimally fitting the calculated fluxes to the direct protein expression data (Østergaard et al., 2016) measured in *Ct*.

**TABLE 6:** Candidate genes for RT-qPCR validation.

**CT no.**	**Gene**	**Reaction^∗∗^**	**EC no.**	**Annotation**	**Pathway^∗∗∗^**
CT121^∗^	*araD*	R01529	5.1.3.1	Ribulose-phosphate 3-epimerase	PPP
CT750^∗^	*tktB*	R01830	2.2.1.1	Transketolase	
CT313^∗^	*tal*	R01827	2.2.1.2	Transaldolase	
CT378^∗^	*pgi*	R02740	5.3.1.9	glucose-6-phosphate isomerase	EMP
CT205	*pfkA_1*	R04779	2.7.1.11	6-phosphofructokinase	
CT505^∗^	*gapA*	R01061	1.2.1.12	Glyceraldehyde 3-phosphate dehydrogenase	
CT332	*pykF*	R00200	2.7.1.40	Pyruvate kinase	
CT207^∗^	*pfkA_2*	R02073	2.7.1.90	Pyrophosphate-fructose-6-phosphate 1-phosphotransferase	
CT714^∗^	*gpdA*	R00842	1.1.1.94	Glycerol-3-phosphate dehydrogenase (NAD(P)()	GPL
CT807	plsB	R00851	2.3.1.15	Glycerol-3-phosphate O-acyltransferase	
CT811	*plsX*				
CT775	*plsC*	R00241	2.3.1.51	1-acyl-sn-glycerol-3-phosphate acyltransferase	
CT376^∗^	*mdhC*	R00342	1.1.1.37	Malate dehydrogenase	TCA
CT855^∗^	*fumC*	R01082	4.2.1.2	Fumarate hydratase class II	
CT821^∗^	*sucC*	R00405	6.2.1.5	Succinyl-CoA synthetase beta subunit	
CT822^∗^	*sucD*			Succinyl-CoA synthetase alpha subunit	
CT054	*sucA*	–	1.2.4.2	2-oxoglutarate dehydrogenase	
CT055	*sucB*		2.3.1.61	2-oxoglutarate dehydrogenase	
CT238^∗^	*fabD*	R01626	2.3.1.39	Acyl-carrier-protein S-malonyltransferase	FA
CT816	*glmS*	R00768	2.6.1.16	Glucosamine-fructose-6-phosphate aminotransferase	Gln/Glu
CT183	*pyrG*	R00571	6.3.4.2	CTP synthase	
CT614	*folX*	R03504	4.1.2.25	Dihydroneopterin aldolase	Folate
CT613	*folP*	R03067	2.5.1.15	Dihydropteroate synthase	
CT612^∗^	*folA*	R02235	1.5.1.3	Dihydrofolate reductase	

**^∗^This enzyme triggers a reversible reaction. ^∗∗^Reaction according to KEGG Reaction number. ^∗∗∗^PPP, pentose phosphate pathway; EMP, glycolysis; GPL, glycerophospholipid biosynthesis; TCA, tricarboxylic acid cycle; FA, fatty acid biosynthesis; Gln/Glu, converting glutamine to glutamate; Folate, Folate biosynthesis.**

In HeLa229 cells (Figure 7A), most of the enzymes in glycolysis were clearly upregulated at 24 hpi, and down regulated at 48 hpi. The two phosphofructokinases were very active, and *pfkA_2*, functional on reversible reactions, was expressed nearly twofold higher at 24 hpi when compared to *pfkA_1*, which triggers the one-way step in glycolysis. Expressions of glucose-6-phosphate isomerase (*pgi*) and pyruvate kinase (*pykF*) were slightly increased (1.25 and 1.2-fold, respectively). This indicates that glycolysis is used strongly by *Ct* no matter whether EB or RB. In accordance with this, the combined data (EB + RB) show an increase in glycolysis in Figure 3B. Regarding iso-enzymes, we find that from the two different forms of phosphofructokinase available to *Ct*, *pfkA_2* is more active in the glycolysis of *Ct* and it uses diphosphate as substrate. In contrast, the substrate ATP is used by *pfkA_1*, with a different sequence and representing a different phosphofructokinase type. According to these qRT-PCR data, in the GPL pathway, the expression of most of the enzymes for phospholipid biosynthesis was reduced during infection (Figure 7, shown in red), except the 1-acyl-sn-glycerol-3-phosphate acyltransferase (*plsC)*, which increased at 24 hpi.

According to our calculations, the biosynthesis of GPL relies more on the downstream part of glycolysis and acyl-CoA, rather than proceeding directly from GAPDH in the upstream part of glycolysis (Figure 6). Expressions of PPP-related genes were not as intense as those in glycolysis. The expression of L-ribulose-5-phosphate 4-epimerase (*araD*) and transketolase (*tktB*) decreased by nearly 50% from 12 to 24 hpi, while transaldolase (*tal*) was slightly reduced. Expressions of the genes in TCA shows a complex picture of comparative activities: The *sucABCD* exhibited about twofold upregulation at 24 hpi, while fumarate hydratase (*fumC*) and malate dehydrogenase (*mdhC*) decreased at 24 hpi and almost invisibly expressed at 48 hpi. Moreover, the malonyl-CoA-ACP transacylase (*fabD*) was involved in the upstream of fatty acid biosynthesis (Table 3, FA1 (as calculated for pathway P67) and FA2 (modeled pathway P68); Figure 3) and associated with the formation of glycerolipids (Table 3, EMP/GPL (calculated flux in pathway P84); Figure 3). It was slightly downregulated at 24 hpi (Figure 7A). In addition, glutamine-fructose-6-phosphate transaminase (*glmS*) and CTP synthase (*pyrG*) are able to irreversibly convert glutamine to glutamate. *glmS* increased its expression by more than a 1.5-fold change at 24 hpi compared to that at 12 hpi, and it was still very active even at 48 hpi, the lysis phase. *pyrG* presented around 50% activity in both 24 and 48 hpi when compared to the 12 hpi. We assume that both of the enzymes may be actively functional in the transfer of glutamine to glutamate. *glmS* could serve for both EB and RB, while *pyrG* is mainly activated in RBs rather than EBs. Three genes in folate biosynthesis were validated. They were significantly decreased by even more than 50% in both 24 and 48 hpi.

The human umbilical vein endothelial cells (HUVECs) were also used as the host for *Ct* infection to make a comparison (Figure 7B). Target genes generally exhibited more expression (and also more deviations) in HUVECs than in HeLa229. Glycolysis, the PPP, and TCA in HUVECs presented similar trends of expression when compared to these pathways in HeLa229. In glycolysis, expression of *pfkA_2* was twofold higher than that of *pfkA_1* at 24 hpi, and threefold higher at 24 hpi compared to 36 hpi. Unlike in HeLa229, glyceraldehyde 3-phosphate dehydrogenase (*gapA*) was upregulated in HUVECs and expressed two times more at 24 hpi than at 12 hpi. In the PPP, the transketolases *tktB* and *tal* showed almost the same expression, but the ribulose-phosphate 3-epimerase (*araD*) was slightly upregulated in HUVECs. In TCA, the succinyl-CoA synthetase (*suc*) genes were overexpressed in both 24 hpi and 36 hpi when compared to those in HeLa229. The other genes involved in glycerophospholipid biosynthesis, fatty acid biosynthesis, glutamine/glutamate transformation, and folate biosynthesis, showed different regulation in HUVECs than in HeLa229. The glycerol-3-phosphate acyltransferases *plsB*, *plsC*, *plsX* together with the acyl-carrier-protein S-malonyltransferase (*fabD*) highly increased the regulation at 24hpi and 36hpi, which suggested the intensive activity of synthesizing glycerophospholipid in HUVECs. The active biosynthesis was also visible by the increased regulation of the dihydrofolate reductase (*folA*), the dihydropteroate synthase (*folP*), and the dihydroneopterin aldolase (*folX*) for folate synthesis, which were differential expressions compared to those in HeLa229. In addition, the expression of *glmS* was similar in both cell lines. However, *pyrG*, which was downregulated in HeLa229, displayed twofold higher upregulation at both 24 and 36 hpi in HUVECs. This suggests the *Ct*’s *pyrG* may be able to convert glutamine to glutamate as well as *glmS.*

## Discussion

### Constraint-Based Metabolic Modeling Enables to Study *Ct* Metabolism During Infection

In this work, *Ct*’s genome-scale metabolic network was first reconstructed based on the constraints, with 321 unique metabolites, 171 enzymes and 277 reactions. Many efforts have been made to elucidate the metabolic properties of *Chlamydia trachomatis* developmental forms during its biphasic life cycle (Saka et al., 2011; Omsland et al., 2012; Käding et al., 2014; Østergaard et al., 2016). Konig et al. (2017) reported the biphasic metabolism of a chlamydial symbiont, the *Protochlamydia amoebophila* UWE25 whose host is *Acanthamoeba*, by using an RNA-Sequencing approach. For the first time, our work compared the differences between EB and RB by quantitative pathways and not only based on the view of expressed genes or gene clusters. According to our results, both EBs and RBs are metabolically dynamic. Their metabolic differentiations are highly variable based on adaptation to the human host environment. With the support of the omics data, the network analysis helps to give an overview of *Ct*’s metabolism according to quantitative pathways. The network was calculated to have 84 pathways and modeled for both EB and RB in the time points of 20 hpi, 40 hpi and the lysis phase respectively. According to our analysis, EB is almost static compared to RB and has its activities centered on the PPP, glycolysis and GPL biosynthesis. Tricarboxylic acid cycle and fatty acid biosynthesis are relatively ineffective. The downstream part of glycolysis and upstream part of fatty acid biosynthesis contribute an efficient metabolic flux to phospholipid metabolism. Enzymes involved in nucleic acid metabolism are strongly expressed in RB, which may offer necessary GTP and ATP for folate biosynthesis and amino acid transformation. EB and RB both have folate biosynthesis pathway active, which utilizes glutamate and PABA to form folate and glycolaldehyde. An overall more intensive flux is present in RB than EB. There is more ATP and GTP available in RB, imported from the host or generated by purine and pyrimidine metabolism.

Genome-scale metabolic modeling is a useful method for microbial engineering and studying pathogenic metabolism. From single organism such as *Mycoplasma genitalium* (Suthers et al., 2009) and Methicillin-resistant *Staphylococcus aureus* (Choe et al., 2018) up to microbial communities (Thiele et al., 2013), metabolic modeling offers a new option to take a quantitative approach to the analysis of complex biological systems via the chemical and mathematical calculation of stoichiometric matrix.

### How Active Are Elementary Bodies?

*Chlamydia* are active in infection. This is self-evident, however, the infective EBs are much less metabolically active than the more active replicating RBs. Extracellular EB’s metabolic activity was detected by Raman micro-spectroscopy (Haider et al., 2010). Omsland et al. (2012) also found the host-free EB could respond to specific metabolites. Nevertheless, the proteomics data by Østergaard et al. (2016) suggest that there is some protein expression also in EB, though less than in RB. An important limitation of extreme pathway modeling based flux calculations is that the activities are only relative and calculated within one organism. Hence, the “stronger” activity for any pathway in EB is only relative to other pathways and the residual activity could be quite low. We provide new data from qRT-PCR in order to find out how active EBs are, however, these data apply only to mRNA expression and not to direct enzyme activity. Nevertheless, this is in agreement to what has been observed in recent studies, Grieshaber et al. (2018) show that generally EBs have lower activity compared to RBs. However, they provide unequivocal proof that there is clearly some metabolic activity in EB and they could detect protein synthesis and active translation of mRNA in EB.

### Chlamydia Metabolism Adapts to Different Cell Lines

The famous HeLa229 cell line is frequently used as the host of *Ct* infection. This tumor-derived cell line has a high metabolic rate and a high frequency in cell division. There are many limitations in gene expression studies with HeLa cell lines. It has been noted that some metabolic activities are influenced by the host cell (Stephens, 1999; Tan and Bavoil, 2012). When in HeLa cell lines, *Ct*’s TCA cycle is supposed to be highly downregulated and nucleic acid metabolism is particularly enriched. The observed TCA cycle, purine, and pyrimidine metabolism is also probably due to the influence of the host HeLa cells. Hence, for comparison, the human umbilical vein endothelial cells (HUVECs) were used as the host for *Ct* infection. *Ct* generally showed more upregulated gene expressions in HUVECs than in HeLa229 cells, especially in the biosynthesis of glycerophospholipid and folate (*pls* and *fol*).

The different gene expressions in different cell lines drive unstable and unpredictable deviations especially in studying pathogen metabolism. Whether the previous metabolic observations (Käding et al., 2014) in HeLa229 cell lines reveal the real metabolism of *Ct* in a natural infection is still an open question. Nevertheless, one approach to improve the metabolic study of *Ct* infection is to use a 3D human tissue model instead of cell lines. A 3D tissue model restores more appropriately the environment for studying the infection of an obligate human pathogen, and offers safer and more efficient support for therapeutic strategies and drug design in the future when compared to animal experiments or human cell lines.

### Carbon and Nitrogen Source Uptake and Energy Production

*Ct* lacks a clear transcriptome response during adaptive change if the carbon source is switched from glucose to glutamate or α-ketoglutarate (Nicholson et al., 2004). Gluconeogenesis is important for regenerating glucose in *Ct* when it encounters different substrates. However, gluconeogenesis is also a process with high energy cost. *Ct* may acquire ATP from the host cell by ATP/ADP transporters and is also able to generate ATP itself according to genome and proteome data. Another important metabolite is glutamate from the host cell. Glutamate is involved in generating NADH, and involves ATP or GTP for triggering folate biosynthesis from the incomplete TCA cycle in *Ct*.

But where does the glutamate come from? Does *Ct* directly take glutamate from the host by glutamate transporter, or does it take glutamine by glutamine transporter, and then converts glutamine to glutamate by enzymes? Three glutamate transporters (CT_216, CT_230 and CT_401) are encoded in the genome (Østergaard et al., 2016). Iliffe-Lee and McClarty (2000) observed the glutamate transporter *gltT* (CT_401) in *Ct*’s carbon metabolism, however, transport of glutamine is still unclear. On the other hand, two enzymes encoded by gene *glmS* and *pyrG* provide capabilities of utilizing glutamine to transfer to glutamate in both cell lines of HeLa229 and HUVECs (Figure 7). *glmS* is upregulated nearly twofold in both cell lines from 12 to 24 hpi, and it is still active in the late lytic phases. pyrG is downregulated in HeLa229 cell lines during infection but comparatively upregulated in HUVECs. Moreover, the CTP synthase, product of *pyrG*, is functionally transcribed and translated during the mid and late stage of the *Ct*’s life cycle (Wylie et al., 1996). According to genome information (Stephens et al., 1998) and previous observations (Weiss, 1967), *Ct* transporters preferentially take up glutamate – not glutamine (Østergaard et al., 2016). What should not be ignored is that the *Ct*-infected host cell lacks an adaptive carbon catabolite repression when the carbon source changes from glucose to glutamate (Nicholson et al., 2004). Taken together, *Ct*’s utilization of glutamate seems to be dependent on the conversion of glutamine by *glmS* and *pyrG*, and may also profit from the intra and extra-cellular environment *via* its transporters.

Our pathway modeling shows that Chlamydial pathways generate energy rich compounds (e.g., NADH and NADPH). This includes glycolysis/gluconeogenesis (P5), folate biosynthesis (P33 and P51), glycerophospholipid biosynthesis (P83), and PPP (P84). According to the available pathways, *Ct* has quite flexible metabolic capabilities, not only between different forms but also with regard to different carbon source utilization. The exact mechanisms of the adaptation still need further study. What we can confirm is that both EB and RB are differentially metabolically active.

### Kinases and Intermediates in Central Metabolism

Two phosphofructokinases are involved in glycolysis, 6-phosphofructokinase 1 (EC 2.7.1.11), and diphosphate-fructose-6-phosphate 1-phosphotransferase (EC 2.7.1.90). These are encoded by *pfkA_1* (CT_205) and *pfkA_2* (CT_207) respectively. The PfkA_2 is similar to PfkA_1, but utilizes diphosphate instead of ATP to catalyze a reversible reaction. Pfk_2 has been found in higher plants, some other eukaryotes, bacteria, and archaea, however, not in *Homo sapiens*. In human cells, this phosphorylation reaction is catalyzed by fructose-1,6-bisphosphatase (EC 3.1.3.11) instead. Hence, *Ct*’s PfkA_2 may be a good drug target for treating *Ct* infection, as this enzyme is not present in humans.

The expression of 1,6-fructose biphosphate aldolase (EC 4.1.2.13) is also able (aldolase B) to reversibly exchange fructose 1-phosphate (F1P) with dihydroxyacetone-P (old name: glycerone-P) and glyceraldehyde. However, due to the lack of fructose kinase in *Ct*, F1P cannot be transformed from fructose. Instead, F1P could possibly be generated as an intermediate in glycolysis, though it is unclear whether the 1,6-fructose bisphosphate aldolase will supply this. A similar situation concerns ribose 1-phosphate produced by ribose 5-phosphate isomerase A (EC 5.3.1.6) which is encoded by *rpiA* (CT_213). Whether F1P and R1P exist in *Ct* and what their functions are is still an open question.

How general are our findings? Well, HeLa229 cells are transformed cervical epithelial cells, while Huvecs are primary endothelial cells derived from human umbilical cord. We tested infection of both these cell lines with *Chlamydia trachomatis* and measured different time points of the infection. The differences in the gene expression show the adaptability of the pathogen depending on the availability of the metabolites and these conclusions should be general for different serovars. Although other serovars have a different genome, their metabolic genes are highly conserved, and so is the mode of intracellular parasitism in this respect. Hence, it is reasonable to expect for these similar results regarding metabolism.

## Conclusion

We have described a first model of *Ct* metabolism after meticulous curation of all available data (proteomics, qRT-PCR data). However, there are no direct metabolite or metabolomics data available. The model takes all extreme pathways accessible for *Ct* into account. It infers detailed information on the pathway changes during infection including differences between EB and RB. *Ct*’s major active pathways are glycolysis, gluconeogenesis, GPL biosynthesis (support from host acetyl-CoA), and the PPP, while its incomplete TCA and fatty acid biosynthesis are inferred to be less active. The modeled metabolic pathways are much more active in RB than in EB. EB and RB utilize folate to generate NAD(P)H using independent pathways. The only low metabolic activity of EB involves the carbohydrate metabolism. RB utilizes energy rich compounds to generate ATP using nucleic acid metabolism enzymes. Experimental data to infer the metabolic model include proteomics experiments (model basis) as well as RT-PCR confirmation of selected enzyme mRNA expression differences at three time points of infection. The metabolic model delivers detailed insights into *Ct* metabolic adaptations during infection and is made available for detailed studies in Chlamydia infection biology.

## Data Availability Statement

All datasets generated for this study are included in the manuscript/Supplementary Files.

## Author Contributions

MY did all the bioinformatics analyses, supervised by TD. KR did all the experiments with initial input from MY. TR provided experimental expert advice. TD provided bioinformatics expert advice. TD led and guided the study. MY and TD drafted the manuscript. All authors participated in the data analysis, refinement of the data (experiments, modeling), and manuscript writing, and agreed to the final version of the manuscript.

## Conflict of Interest

The authors declare that the research was conducted in the absence of any commercial or financial relationships that could be construed as a potential conflict of interest.

## Footnotes

1 http://proteomecentral.proteomexchange.org

## Supplementary Material

FILE S1 Genes and reactions with annotations used in the construction of the metabolic model. It includes the gene names of *Ct*, reaction numbers according to KEGG, EC numbers, reversibility of the reaction, and all annotations.

FILE S2 *Ct* metabolic flux model in SBML (level2) format. This model was next imported into YANAsquare to calculate metabolic fluxes.

FILE S3 Null space matrix of the *Ct* metabolic flux model.

FILE S4 Stoichiometry matrix of the *Ct*’s metabolic flux model.

FILE S5 Results of all extreme pathways calculated by *Ct*’s model with data import of proteome from Østergaard et al. (2016). It shows pathway strengths quantitatively. Positive or negative signs in the values show the direction of the reaction. Positive values indicate a forward reaction, and negative values show that the reaction goes in the reverse direction. EB1, EB2, and EB3 show the calculated pathway strengths in elementary bodies at 20 hpi, 40 hpi and after 40 hpi, respectively. RB1, RB2, and RB3 provide the same information but for reticulate bodies. The EBRB values EBRB1-3 are calculated using the sum of the proteomic values of EB and RB as data import, this calculation infers the general metabolic pathway strengths of *Ct*. The average strength is marked by the “AVE” tag.

FILE S6 Steady state calculated from the reconstructed model, showing all internal and external metabolites and flux strengths quantitatively. Internal and external metabolites are shown by black balls and red cones, respectively. The pathway with the strongest flux is shown as blue thick line (path from pyruvate→acetyl-CoA→malonyl-CoA = malonyl-ACP).

FILE S7 Pathways differentially expressed in *Ct*’s different forms during infection. Detailed data for the *Ct* mixture during infection (EB and RB) as well as for EB alone and RB alone are given and color coded according to flux intensity. Early infection is always shown at the bottom, late infection shown at the top. Shown are flux strengths for three time points: at 20 hpi (hours post infection), 40 hpi and approximately 8 hours later (40 hpi post, see section “Materials and Methods”). The bar on top illustrates the color code: maximum intensity (dark blue) is 1, minimal intensity (white) is zero. Major extreme pathways inferred involve the strongest flux path from pyruvate to malonyl-ACP (P65), followed by the downstream of glycolysis (P5, P34, and P83), upstream of fatty acid biosynthesis (P66 and P67) and involved in biotin metabolism (P65). Cysteine biosynthesis also is inferred to have a strong flux (P16). Many reactions in nucleotide metabolism are calculated to have strong intensities (P8–P15, P31, P32, and P40–45). Only medium activity was inferred for glycolysis and glycerophospholipid pathway (P34 and P83), pentose phosphate pathway (P84) and folate biosynthesis or conversion (P33, P51, and P55). Very low activity at all times was inferred for fatty acid biosynthesis (P66 and P67), phenylalanine and tyrosine biosynthesis (P53 and P54), as well as porphyrin and chlorophyll metabolism (P29).

## References

[B1] Abdelrahman Y. M. Belland R. J. (2005). The chlamydial developmental cycle. *FEMS Microbiol. Rev.* 29 949–959. 16043254 10.1016/j.femsre.2005.03.002

[B2] Adams N. E. Thiaville J. J. Proestos J. Juarez-Vazquez A. L. McCoy A. J. Barona-Gomez F. (2014). Promiscuous and adaptable enzymes fill “holes” in the tetrahydrofolate pathway in *Chlamydia* species. *mBio* 5:e01378-14. 10.1128/mBio.01378-14 25006229 PMC4161248

[B3] Albrecht M. Sharma C. M. Reinhardt R. Vogel J. Rudel T. (2010). Deep sequencing-based discovery of the *Chlamydia trachomatis* transcriptome. *Nucleic Acids Res.* 38 868–877. 10.1093/nar/gkp1032 19923228 PMC2817459

[B4] Belland R. J. Zhong G. Crane D. D. Hogan D. Sturdevant D. Sharma J. (2003). Genomic transcriptional profiling of the developmental cycle of *Chlamydia trachomatis*. *Proc. Natl. Acad. Sci. U.S.A.* 100 8478–8483. 10.1073/pnas.1331135100 12815105 PMC166254

[B5] Betts-Hampikian H. J. Fields K. A. (2011). Disulfide bonding within components of the *Chlamydia* Type III secretion apparatus correlates with development. *J. Bacteriol.* 193 6950–6959. 10.1128/JB.05163-11 22001510 PMC3232835

[B6] Chesson H. W. Pinkerton S. D. (2000). Sexually transmitted diseases and the increased risk for HIV transmission: implications for cost-effectiveness analyses of sexually transmitted disease prevention interventions. *J. Acquir. Immune Defic. Syndr.* 24 48–56. 10.1097/00042560-200005010-00009 10877495

[B7] Choe D. Szubin R. Dahesh S. Cho S. Nizet V. Palsson B. (2018). Genome-scale analysis of Methicillin-resistant *Staphylococcus aureus* USA300 reveals a tradeoff between pathogenesis and drug resistance. *Sci. Rep.* 8:2215. 10.1038/s41598-018-20661-1 29396540 PMC5797083

[B8] Cocchiaro J. L. Kumar Y. Fischer E. R. Hackstadt T. Valdivia R. H. (2008). Cytoplasmic lipid droplets are translocated into the lumen of the *Chlamydia trachomatis* parasitophorous vacuole. *Proc. Natl. Acad. Sci. U.S.A.* 105 9379–9384. 10.1073/pnas.0712241105 18591669 PMC2453745

[B9] Fan H. Brunham R. C. McClarty G. (1992). Acquisition and synthesis of folates by obligate intracellular bacteria of the genus *Chlamydia*. *J. Clin. Invest.* 90 1803–1811. 10.1172/jci116055 1430206 PMC443239

[B10] Grieshaber S. Grieshaber N. Yang H. Baxter B. Hackstadt T. Omsland A. (2018). Impact of active metabolism on *Chlamydia trachomatis* elementary body transcript profile and infectivity. *J. Bacteriol.* 200:e00065-18. 10.1128/JB.00065-18 29735758 PMC6018357

[B11] Haider S. Wagner M. Schmid M. C. Sixt B. S. Christian J. G. Hacker G. (2010). Raman microspectroscopy reveals long-term extracellular activity of *Chlamydiae*. *Mol. Microbiol.* 77 687–700. 10.1111/j.1365-2958.2010.07241.x 20545842

[B12] Harris S. R. Clarke I. N. Seth-Smith H. M. Solomon A. W. Cutcliffe L. T. Marsh P. (2012). Whole-genome analysis of diverse *Chlamydia trachomatis* strains identifies phylogenetic relationships masked by current clinical typing. *Nat. Genet.* 44 413–419. 10.1038/ng.2214 22406642 PMC3378690

[B13] Hove-Jensen B. Andersen K. R. Kilstrup M. Martinussen J. Switzer R. L. Willemoes M. (2017). Phosphoribosyl diphosphate (PRPP): biosynthesis, enzymology, utilization, and metabolic significance. *Microbiol. Mol. Biol. Rev.* 81 e00040-16. 10.1128/MMBR.00040-16 28031352 PMC5312242

[B14] Iliffe-Lee E. R. McClarty G. (2000). Regulation of carbon metabolism in *Chlamydia trachomatis*. *Mol. Microbiol.* 38 20–30. 10.1046/j.1365-2958.2000.02102.x 11029687

[B15] Käding N. Szaszak M. Rupp J. (2014). Imaging of *Chlamydia* and host cell metabolism. *Future Microbiol.* 9 509–521. 10.2217/fmb.14.13 24810350

[B16] Kaleta C. Centler F. Dittrich P. (2006). Analyzing molecular reaction networks: from pathways to chemical organizations. *Mol. Biotechnol.* 34 117–123. 17172657 10.1385/MB:34:2:117

[B17] Kanehisa M. Goto S. (2000). KEGG: kyoto encyclopedia of genes and genomes. *Nucleic Acids Res.* 28 27–30. 10.1093/nar/28.1.27 10592173 PMC102409

[B18] Karunakaran K. Subbarayal P. Vollmuth N. Rudel T. (2015). *Chlamydia*-infected cells shed Gp96 to prevent *chlamydial* re-infection. *Mol. Microbiol.* 98 694–711. 10.1111/mmi.13151 26235316

[B19] Konig L. Siegl A. Penz T. Haider S. Wentrup C. Polzin J. (2017). Biphasic metabolism and host interaction of a *Chlamydial* symbiont. *mSystems* 2:e00202-16. 10.1128/mSystems.00202-16 28593198 PMC5451489

[B20] Kumar Y. Cocchiaro J. Valdivia R. H. (2006). The obligate intracellular pathogen *Chlamydia trachomatis* targets host lipid droplets. *Curr. Biol.* 16 1646–1651. 10.1016/j.cub.2006.06.060 16920627

[B21] Liechti G. Kuru E. Packiam M. Hsu Y. P. Tekkam S. Hall E. (2016). Pathogenic *Chlamydia* lack a classical sacculus but synthesize a narrow. *PLoS Pathog.* 12:e1005590. 10.1371/journal.ppat.1005590 27144308 PMC4856321

[B22] Liechti G. W. Kuru E. Hall E. Kalinda A. Brun Y. V. VanNieuwenhze M. (2014). A new metabolic cell-wall labelling method reveals peptidoglycan in *Chlamydia trachomatis*. *Nature* 506 507–510. 10.1038/nature12892 24336210 PMC3997218

[B23] Mehlitz A. Eylert E. Huber C. Lindner B. Vollmuth N. Karunakaran K. (2017). Metabolic adaptation of *Chlamydia trachomatis* to mammalian host cells. *Mol. Microbiol.* 103 1004–1019. 10.1111/mmi.13603 27997721

[B24] Nicholson T. L. Chiu K. Stephens R. S. (2004). *Chlamydia trachomatis* lacks an adaptive response to changes in carbon source availability. *Infect. Immun.* 72 4286–4289. 10.1128/iai.72.7.4286-4289.2004 15213176 PMC427450

[B25] Omsland A. Sager J. Nair V. Sturdevant D. E. Hackstadt T. (2012). Developmental stage-specific metabolic and transcriptional activity of *Chlamydia trachomatis* in an axenic medium. *Proc. Natl. Acad. Sci. U.S.A* 109 19781–19785. 10.1073/pnas.1212831109 23129646 PMC3511728

[B26] Orth J. D. Thiele I. Palsson B. Ø (2010). What is flux balance analysis. *Nat. Biotechno.l* 28 245–248. 10.1038/nbt.1614 20212490 PMC3108565

[B27] Østergaard O. Follmann F. Olsen A. W. Heegaard N. H. Andersen P. Rosenkrands I. (2016). Quantitative protein profiling of *Chlamydia trachomatis* growth forms reveals defense strategies against tryptophan starvation. *Mol. Cell Proteomics* 15 3540–3550. 10.1074/mcp.m116.061986 27784728 PMC5141270

[B28] Pfeiffer T. Sanchez-Valdenebro I. Nuno J. C. Montero F. Schuster S. (1999). METATOOL: for studying metabolic networks. *Bioinformatics* 15 251–257. 10.1093/bioinformatics/15.3.251 10222413

[B29] Pilhofer M. Aistleitner K. Biboy J. Gray J. Kuru E. Hall E. (2013). Discovery of *Chlamydial* peptidoglycan reveals bacteria with murein sacculi but without FtsZ. *Nat. Commun.* 4:2856. 10.1038/ncomms3856 24292151 PMC3847603

[B30] Read T. D. Brunham R. C. Shen C. Gill S. R. Heidelberg J. F. White O. (2000). Genome sequences of *Chlamydia trachomatis* MoPn and *Chlamydia* pneumoniae AR39. *Nucleic Acids Res.* 28 1397–1406. 10.1093/nar/28.6.1397 10684935 PMC111046

[B31] Saka H. A. Thompson J. W. Chen Y. S. Kumar Y. Dubois L. G. Moseley M. A. (2011). Quantitative proteomics reveals metabolic and pathogenic properties of *Chlamydia trachomatis* developmental forms. *Mol. Microbiol.* 82 1185–1203. 10.1111/j.1365-2958.2011.07877.x 22014092 PMC3225693

[B32] Schomburg I. Chang A. Schomburg D. (2002). BRENDA, enzyme data and metabolic information. *Nucleic Acids Res.* 30 47–49. 10.1093/nar/30.1.47 11752250 PMC99121

[B33] Schwarz R. Liang C. Kaleta C. Kuhnel M. Hoffmann E. Kuznetsov S. (2007). Integrated network reconstruction, visualization and analysis using YANAsquare. *BMC Bioinform.* 8:313. 10.1186/1471-2105-8-313 17725829 PMC2020486

[B34] Senior K. (2012). *Chlamydia*: a much underestimated STI. *Lancet Infect Dis* 12 517–518. 10.1016/s1473-3099(12)70161-5 22930827

[B35] Skipp P. J. Hughes C. McKenna T. Edwards R. Langridge J. Thomson N. R. (2016). Quantitative Proteomics of the infectious and replicative forms of *Chlamydia trachomatis*. *PLoS One* 11:e0149011. 10.1371/journal.pone.0149011 26871455 PMC4752267

[B36] Stephens R. S. (1999). *Chlamydia: Intracellular Biology, Pathogenesis, and Immunity.* Grand Rapids: Zondervan.

[B37] Stephens R. S. Kalman S. Lammel C. Fan J. Marathe R. Aravind L. (1998). Genome sequence of an obligate intracellular pathogen of humans: *Chlamydia trachomatis*. *Science* 282 754–759. 10.1126/science.282.5389.754 9784136

[B38] Subtil A. Dautry-Varsat A. (2004). *Chlamydia*: five years A.G. (after genome). *Curr. Opin. Microbiol.* 7 85–92. 10.1016/j.mib.2003.12.012 15036146

[B39] Suthers P. F. Dasika M. S. Kumar V. S. Denisov G. Glass J. I. Maranas C. D. (2009). A genome-scale metabolic reconstruction of *Mycoplasma genitalium*, iPS189. *PLoS Comput. Biol.* 5:e1000285. 10.1371/journal.pcbi.1000285 19214212 PMC2633051

[B40] Tan M. Bavoil P. (2012). *Intracellular Pathogens I: Chlamydiales*, Vol. 1 Washington, DC: American Society for Microbiology Press.

[B41] Thiele I. Swainston N. Fleming R. M. Hoppe A. Sahoo S. Aurich M. K. (2013). A community-driven global reconstruction of human metabolism. *Nat. Biotechnol.* 31 419–425. 10.1038/nbt.2488 23455439 PMC3856361

[B42] von Kamp A. Schuster S. (2006). Metatool 5.0: fast and flexible elementary modes analysis. *Bioinformatics* 22 1930–1931. 10.1093/bioinformatics/btl267 16731697

[B43] Weiss E. (1967). Transaminase activity and other enzymatic reactions involving pyruvate and glutamate in *Chlamydia* (psittacosis-trachoma group). *J. Bacteriol.* 93 177–184. 6020405 10.1128/jb.93.1.177-184.1967 PMC314986

[B44] Wylie J. L. Berry J. D. McClarty G. (1996). *Chlamydia trachomatis* CTP synthetase: molecular characterization and developmental regulation of expression. *Mol. Microbiol.* 22 631–642. 10.1046/j.1365-2958.1996.d01-1717.x 8951811

[B45] Xie G. Bonner C. A. Jensen R. A. (2002). Dynamic diversity of the tryptophan pathway in *Chlamydiae*: reductive evolution and a novel operon for tryptophan recapture. *Genome Biol.* 3 research0051.1–research0051.17. 12225590 10.1186/gb-2002-3-9-research0051 PMC126876

[B46] Yao J. Dodson V. J. Frank M. W. Rock C. O. (2015). *Chlamydia trachomatis* scavenges host fatty acids for phospholipid synthesis via an Acyl-Acyl carrier protein synthetase. *J. Biol. Chem.* 290 22163–22173. 10.1074/jbc.M115.671008 26195634 PMC4571967

