# Supplementary figures and images for: Comprehensive Flux Modeling of Chlamydia trachomatis Proteome and qRT-PCR Data Indicate Biphasic Metabolic Differences Between Elementary Bodies and Reticulate Bodies During Infection
Source: Front Microbiol. 2019 Oct 15;10:2350. doi: 10.3389/fmicb.2019.02350 (PMC6803457; doi:10.3389/fmicb.2019.02350)

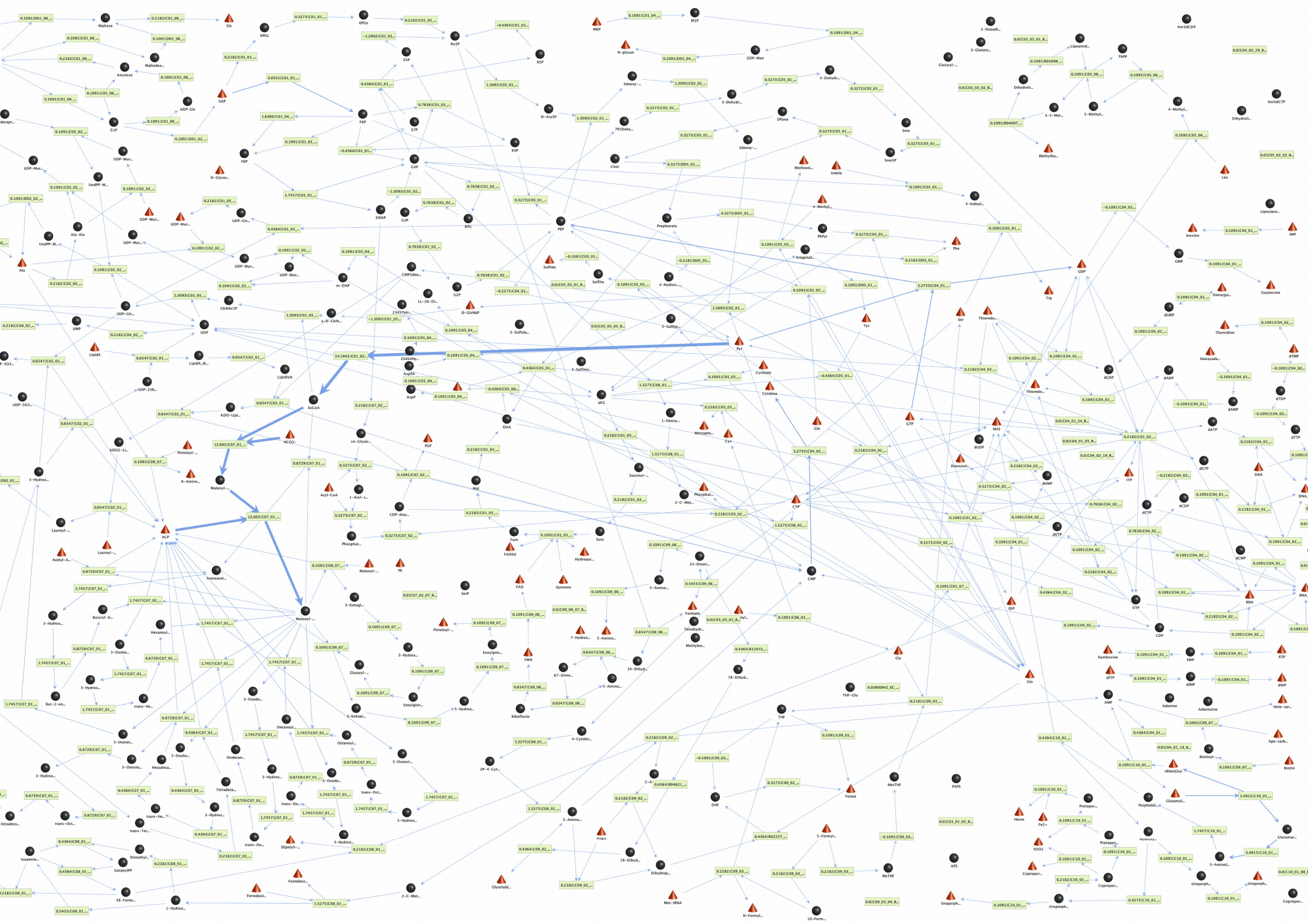

Supplement: FILE S1 — Genes and reactions with annotations used in the construction of the metabolic model. It includes the gene names of Ct, reaction numbers according to KEGG, EC numbers, reversibility of the reaction, and all annotations. [file Data_Sheet_1.ZIP › S6_FluxNetwork.pdf]

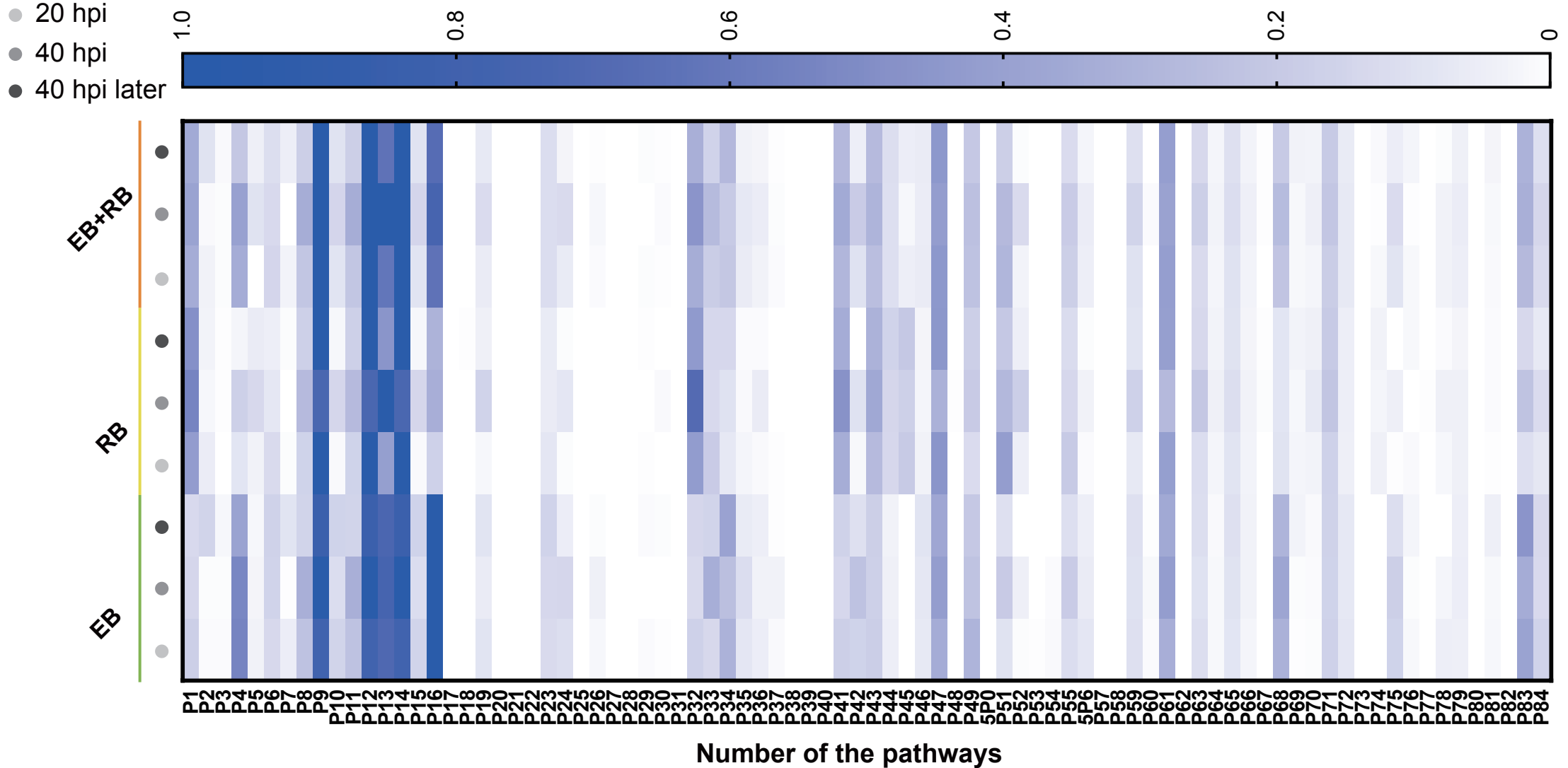

Supplement: FILE S1 — Genes and reactions with annotations used in the construction of the metabolic model. It includes the gene names of Ct, reaction numbers according to KEGG, EC numbers, reversibility of the reaction, and all annotations. [file Data_Sheet_1.ZIP › S7_all_extreme_pathways.pdf]
